# The impact of (poly)phenol-rich sugarcane extract intervention on markers of gastrointestinal integrity and systemic inflammation in response to exertional-heat stress

**DOI:** 10.1080/15502783.2026.2684574

**Published:** 2026-06-18

**Authors:** Ulluwis H. A. J Hewawansa, Kayla Henningsen, Natália Vilela Silva Daniel, Sandra In, Giles Sugay, Michael J. Houghton, Elizabeth Barber, Gary Williamson, Ricardo J. S. Costa

**Affiliations:** a Department of Nutrition Dietetics & Food, Faculty of Medicine, Nursing and Health Sciences, Monash University, Notting Hill, VIC, Australia; b Victorian Heart Institute, Faculty of Medicine, Nursing and Health Sciences, Monash University, Clayton, VIC, Australia; c Faculdade de Ciências Aplicadas, Universidade Estadual de Campinas (FCA/UNICAMP), Limeira, São Paulo, Brazil; d School of Biological Sciences, Queen's University, Belfast, UK

**Keywords:** Exercise-induced gastrointestinal syndrome, gastrointestinal symptoms, I-FABP, endotoxin, running, polyphenol

## Abstract

**Background:**

Exertional-heat stress (EHS) perturbs gastrointestinal integrity, leading to systemic inflammation that may warrant medical attention. The study aimed to determine the effects of a (poly)phenol-rich sugarcane extract (PRSE) beverage and gel interventions on biomarkers of intestinal epithelial integrity and systemic immune responses to an EHS challenge.

**Method:**

Using a double-blind placebo-controlled design, participants (*n* = 14) were randomly allocated to complete two EHS (i.e. 2 h running at 60% $\dot{V}O_{2\max}$ in 34.4 °C ambient temperature) trials, with at least one-week washout. Participants consumed 2 × 270 mL beverages daily for 14 days before EHS, and 1 × 270 mL beverage pre-EHS and a gel sachet every 20 min during EHS. Beverages and gels were either PRSE (beverage: 10% w/v, gel: 46% w/v) or carbohydrate-matched placebo. Whole blood samples were collected pre-EHS, immediately post-EHS and 30 min, 1 h, and 2 h post-EHS, and analyzed for plasma concentrations of cortisol, I-FABP, sCD14, and systemic inflammatory cytokines.

**Results:**

Compared with placebo, the PRSE trial exhibited higher rectal temperature (*p* = 0.046) and physiological strain index (*p* = 0.010). No Trial × Time interaction was observed for I-FABP; however, pre-EHS plasma I-FABP concentration was lower in PRSE compared to placebo. Plasma IL-8 concentration increased over time (*p* < 0.001) that was attenuated by PRSE compared with placebo (*p* = 0.023). Exercise-associated gastrointestinal symptoms (Ex-GIS) incidence was high in both trials, while average symptom severity was lower with PRSE.

**Conclusion:**

Two weeks of PRSE supplementation with a carbohydrate-containing beverage during EHS may provide modest advantages to intestinal epithelial integrity and systemic inflammatory, without further exacerbating Ex-GIS.

## 1. Introduction

Exogenous carbohydrate supplementation during prolonged endurance exercise is well established to support physical performance and delay fatigue [1,2]. As exercise duration increases, skeletal muscle glucose uptake and oxidation increases, particularly as endogenous glycogen stores become depleted [3,4]. Maintenance of blood glucose during prolonged exercise relies on hepatic glucose production via gluconeogenesis and liver glycogenolysis [5]. However, during extended exertion, hepatic glucose output may become insufficient to meet skeletal muscle requirement, resulting in hypoglycemia, and subsequently demanding exogenous carbohydrate intake to maintain euglycemia and exercise capacity [6]. Hypoglycemia during endurance exercise has also been associated with exercise-induced nausea and other exercise-associated gastrointestinal symptoms (Ex-GIS), which can impair performance [3,7]. Consequently, guidelines for prolonged endurance exercise (≥3 h) recommend carbohydrate intakes of ~30–90 g·h^−1^, adjusted for individual tolerance and feasibility [1,7]. Given marked interindividual variability in feeding tolerance, glucose availability, and carbohydrate oxidation [4,7,8], there is a need for diverse nutritional strategies to meet individual requirements.

Alongside carbohydrate provision, maintaining hydration status to prevent excessive hypohydration is critical, particularly during prolonged exercise in hot ambient conditions where sweat losses are elevated [9–11]. Plasma osmolality range indicative of euhydration typically ranges between 280 and 300 mOsm·kg^−1^ [12,13], and endurance performance is impaired when exercise is performed in a hypohydrated state [14,15]. Emerging evidence suggests that, beyond traditional electrolyte-based approaches, osmotically active nutrients such as carbohydrates and proteins may contribute to body water retention during prolonged exercise, particularly under heat stress conditions [11,16–20].

Exercise-induced gastrointestinal syndrome (EIGS) is a common complication of prolonged endurance exercise and is characterized by gastrointestinal symptoms arising primarily from splanchnic hypoperfusion and increased sympathetic activation, leading to compromised epithelial integrity and impaired gastrointestinal function [7]. These disturbances are exacerbated during exertional heat stress (EHS), when elevated core and skin temperatures increase cardiovascular and thermoregulatory strain and promote redistribution of cardiac output toward the skin, further reducing splanchnic blood flow and gastrointestinal oxygen delivery [21]. Consequently, exercise in hot environments (e.g. 2 h steady-state running at ~35 °C ambient temperature) is associated with greater gastrointestinal epithelial injury, increased intestinal permeability, and heightened severity of Ex-GIS compared with temperate conditions [21,22]. At the cellular level, acute intestinal injury during prolonged exercise and EHS activates NF-κB signaling within epithelial cells, initiating a local inflammatory cascade characterized by increased production of proinflammatory cytokines such as IL-1β, and TNF-*α* [23,24]. These cytokines promote tight-junction dysfunction, increased intestinal permeability, and translocation of luminal pathogenic components, thereby amplifying local and systemic inflammatory responses, which in severe cases may progress to clinically significant outcomes [25,26]. Hypohydration and inadequate nutrient intake further exacerbates these mechanisms, whereas frequent feeding during EHS, including carbohydrate, protein, and amino acid provision, has been shown to attenuate gastrointestinal perturbations [26–28].

Beyond macronutrient provision, (poly)phenol-rich nutritional strategies may plausibly confer additional gastrointestinal protection during EHS by targeting key mechanisms underpinning EIGS and Ex-GIS. (Poly)phenols have been shown to attenuate NF-κB-driven inflammatory signaling, reduce proinflammatory cytokine production, and preserve epithelial barrier integrity [29–31]. (Poly)phenol-rich sugarcane extract (PRSE), an extract derived from molasses, also a by-product of sugarcane processing, is rich in phenolic compounds [32] and naturally contains carbohydrates. Preclinical studies have demonstrated anti-inflammatory effects of PRSE [33–35], providing a mechanistic rationale for investigating PRSE as a nutritional strategy to attenuate gastrointestinal epithelial injury and systemic immune disturbances associated with EHS. For instance, chlorogenic acid, a major phenolic acid present in sugarcane [32], has been shown to improve intestinal barrier function and favorably influence gut microbial composition [36], highlighting the relevance of sugarcane-derived (poly)phenols for mitigating EIGS and Ex-GIS under EHS conditions. Therefore, this study aimed to determine the effects of PRSE beverage and gel interventions on intestinal epithelial integrity and systemic immune responses to EHS challenge. It was hypothesized that PRSE supplementation would attenuate intestinal epithelial injury and permeability, systemic endotoxemia and inflammatory responses, without exacerbating Ex-GIS, to an EHS challenge, compared with a carbohydrate-matched placebo.

## 2. Methods

### 2.1. Participants

Fourteen endurance-trained adult athletes, who were not heat-acclimatized, volunteered to participate in the study. Each participant provided written informed consent prior to commencement. The study protocol received approval from the Monash University Human Research Ethics Committee (ID: 31544) and adhered to the ethical standards outlined in the 2013 Declaration of Helsinki. Additionally, this trial was registered with the Australian New Zealand Clinical Trials Registry (ID: 12624000285550). All individuals that enquired to volunteer for the study were screened to assess if they have gastrointestinal infections, diseases, and/or disorders (e.g. celiac disease, inflammatory bowel disease, irritable bowel syndrome, diverticular disease, gastro-esophageal reflux disease, past history of gastrointestinal surgery, and/or other self-identified gastrointestinal issues), consumed potential modifiers of gastrointestinal integrity (i.e. prebiotics, probiotics, postbiotics, synbiotics, and/or antibiotics), adhered to gastrointestinal-focused dietary regimes (i.e. low fermentable oligo, di-, mono-saccharide, and polyol (FODMAP) and/or fiber-modified diets) within the previous 3 months, or consumed nonsteroidal anti-inflammatory medications and/or stool altering medications (i.e. laxatives and/or antidiarrheal) within 1 month before the experimental protocol, and were excluded from the study if they indicated yes to one or more of the gastrointestinal related screening criteria.

### 2.2. Preliminary measures

Approximately a week before the first experimental trial, participants' height was determined by a stadiometer (Holtain Limited, Crosswell, UK) and body composition was measured using an 8-point multi-frequency bioelectrical impedance analyzer (mBCA 515, Seca, Ecomed, Hamburg, Germany). Maximal oxygen uptake ( $\dot{V}O_{2\max}$ ) was assessed through a continuous incremental exercise test to the point of exhaustion on a motorized treadmill (Cosmos treadmill, h/p/Cosmos Sports and Medical GmbH, Nussdorf-Traunstein, Germany) with breath-by-breath indirect calorimetry (Jaëger CPX, Vyntus, Vyaire Medical, Mettawa, IL, USA). To establish the running speed for the exercise trials, the speed corresponding to approximately 60% of $\dot{V}O_{2\max}$ at a 1% gradient was calculated and confirmed based on the $\dot{V}O_{2}$ –work rate relationship.

### 2.3. Intervention and experimental procedure

The clinical intervention used PRSE that was developed into an isotonic sports beverage (10% w/v) and gel (46% w/v) by The Product Makers, Keysborough, VIC, Australia. PRSE is an ethanolic resin-based extraction of molasses, a by-product of the sugarcane industry. The full (poly)phenol composition of PRSE has been published in our previous work [37]. PRSE contains 44.2% total sugars including 32% sucrose, 6.9% fructose, 5.3% glucose (measured by National Measurements Institute in-house method VL295). The placebos were matched to the PRSE beverage and gel, with equivalent sugar but without (poly)phenols. Placebos and PRSE beverages were formulated with identical ion composition using a premixed electrolyte solution (i.e. Na^+^, Cl^−^, K^+^, Mg^2+^, and Ca^2+^), and osmolality was equally adjusted to an isotonic range (250–340 mOsm/L) in accordance with Food Standards Australia New Zealand guidelines.

**Figure 1.**
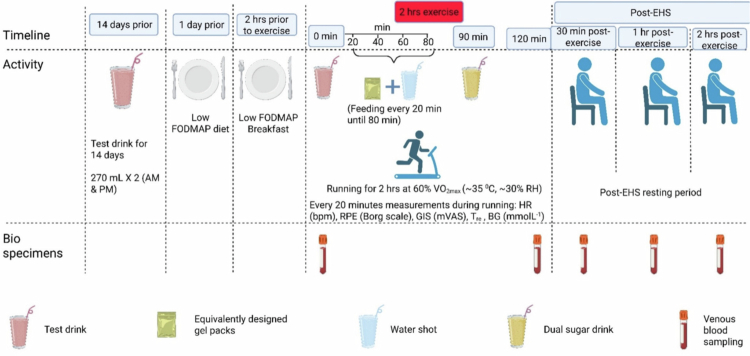
Schematic flow diagram of study design, experimental protocol and sample collection times. EHS = exertional-heat stress; HR = heart rate; RPE = rating of perceived exertion; GIS = Gastrointestinal symptoms; T_re_ = Rectal temperature; BG = Blood glucose; FODMAP = fermentable oligosaccharides, disaccharides, monosaccharides and polyols; mVAS = modified visual analog scale; RH = relative humidity. Created by Biorender (biorender.com).

A schematic overview of the study and its experimental procedures is shown in Figure 1. This study adhered to best practice guidelines for exercise gastroenterology [38]. Following pre-exercise measurements and preparations, participants completed two experimental trials in a double-blind, randomized, and counterbalanced order, separated by at least two weeks. After the 14-days beverage intervention, participants completed the EHS challenge. The EHS trial involved 2 h of running on a motorized treadmill at a speed eliciting 60% $\dot{V}O_{2\max}$ as determined in the initial assessment, within an environmental chamber at 34.4 (1.2) °C ambient temperature and 30.3 (2.5) % relative humidity. The order of the two trials (PRSE and placebo) was randomized using a pick-out-of-the-hat method by a researcher not involved in participant testing (GW). Participants were assigned to receive either PRSE or the matched placebo during the first trial, with the alternate formulation administered during the subsequent trial. Allocation and supplement preparation were performed by another researcher not involved in participant testing (EB). Participants consumed a 270 mL beverage with breakfast and dinner (totaling 540 mL/day) for 14 days prior to the EHS challenge. Participants were provided with a low-FODMAP diet for 24 h [10.87 (0.88) MJ/day, 411 (40) g/day carbohydrate (64.24% energy contribution), 100 (13) g/day protein (15.63% energy contribution), 46 (13) g/day fat (15.65% energy contribution), and 39 (5) g/day fiber] before each experimental trial to minimize dietary confounding factors [39,40]. Compliance was monitored and verified through a standardized intake compliance log. Participants were also instructed to avoid additional high-FODMAP foods, alcohol, and caffeinated beverages during this period, and to refrain from strenuous exercise for 24 h before each trial. Participants arrived at the laboratory at 8:00 AM after consuming a low-FODMAP breakfast [2.5 MJ, 112 g carbohydrate, 16 g protein, 4 g fat, and 10 g fiber] with 500 mL of water at 7:00 AM. Upon arrival, they voided before nude body mass (Seca 813, Seca Group, Hamburg, Germany) and total body water measurements using an 8-point multifrequency bioelectrical impedance analyzer (mBCA 515, Seca, Ecomed, Hamburg, Germany). Blood samples were collected via venipuncture from an antecubital vein into sterile lithium heparin (6 mL, 1.5 IU/mL heparin) and a K3EDTA (4 mL, 1.6 mg/mL EDTA) (Becton Dickinson, Oxford, UK) tubes. To monitor rectal temperature (Tre) during running, participants inserted a thermocouple 12–15 cm beyond the external anal sphincter (Alpha Technics Precision Temperature 4600 Thermometer). On the trial day, they consumed one 270 mL beverage bottle before exercise and equivalent gel packs every 20 min (from 0 to 80 min) during the EHS challenge, as described in Figure 1.

To minimize seasonal heat acclimatization, the experimental procedures were conducted during the cooler months in Melbourne, Victoria, Australia, with temperatures consistently ≤20 °C from May 2024 to October 2024. Heart rate, rating of perceived exertion (RPE), thermal comfort rating (TCR), T_re_, Ex-GIS, ambient temperature, and relative humidity were measured every 20 min during EHS, as previously reported [26,39]. The physiological strain index (PSI) was calculated from relative changes to heart rate and T_re_ that was used to represent the combined strain of the cardiovascular and thermoregulatory systems [41]. Participants ingested a solution consisting of 10 g lactulose (Actilax, Alphapharm Pty Ltd trading as Viatris, Carole Park, QLD 4300, Australia) and 2 g of L-rhamnose (MarkNature, Fullerton, California, USA) in 150 ml of water 90 min into exercise (30 min prior to finish) to assess small intestinal permeability via the dual sugar absorption test [28]. Immediately after the EHS exposure, blood samples were collected, and nude body mass was recorded. Participants remained seated during the recovery period and were provided with water *ad libitum*. Additional blood samples were collected 30  min, 1 h and 2 h after. GIS was also recorded every 20  min during the 2  h recovery period.

### 2.4. Sample analysis

Whole blood hemoglobin (HB201+, HemoCue AB) and hematocrit values obtained via the capillary method were used to estimate changes in plasma volume relative to baseline, which were then used to adjust plasma variables [13,42]. Blood glucose levels were measured before and after EHS using a HemoCue system (Glucose 201 C, HemoCue AB) in duplicate [CV: 3.8%] from heparinized whole blood samples. The remaining blood samples were centrifuged at 2415 × g for 10 min at 4°C. The plasma was aliquoted into microtubes and frozen at −80 °C until analysis, except for 2 × 50 μL used to determine plasma osmolality (P_Osmol_) in duplicate via freeze-point osmometry (Osmomat 030, Gonotec) [CV: 1.0%]. Plasma concentrations of cortisol (ab108665, Abcam), intestinal fatty acid-binding protein (I-FABP; HK406, Hycult Biotech), soluble CD14 (sCD14; HK320, Hycult Biotech), and interleukins (IL-1β, TNF-*α*, IL-8, IL-10, and IL-1ra) were measured by ELISA (Invitrogen, Thermo Fisher Scientific; kit codes: BMS224HS, KHC3014, KHC0084, EHIL1R1). Each plasma variable was analyzed in duplicate in accordance with the manufacturer's instructions, with standards and controls on each plate, and each participant assayed on the same plate. The intra-assay CV for I-FABP, sCD14, TNF-*α*, IL-8, IL-10, and IL-1ra was 6.8%, 3.3%, 4.3%, 3.3%, 9.7%, 3.8%, respectively. Plasma IL-6 concentration was determined using a proximity ligation immunoassay with qPCR-based detection (ProQuantum™ Human IL-6 Immunoassay Kit, A35573, Invitrogen, Thermo Fisher Scientific). The systemic inflammatory response profile (SIR-Profile) was calculated from the combined relative changes of cytokines measures in the current study that was used to represent the combined systemic inflammation, as previously proposed and reported [26,38]. Dual sugars were quantified using high-performance anion exchange chromatography with pulsed amperometric detection (HPAEC-PAD) using a previously established protocol in our lab [28]. The intra-measurement CV for lactulose and L-rhamnose were 11% and 2%.

### 2.5. Statistical analysis

Based on historical data (1619 [1398] pg/mL) for changes in plasma I-FABP concentration in response to EHS [39,43,44] and using standard alpha (0.05) and beta (0.80) values, the current sample size is estimated to provide sufficient statistical power (*n* = 8; power × 0.80–0.99) to detect significant differences between trials (G*Power, version 3.1). The mean and standard deviation [SD] are shown for data in the text and tables. For clarity, mean ± standard error of the mean (SEM) is used to show absolute data in figures, and relative data (*Δ*) are shown as box and whisker plots. All data were checked for normality using skewness and kurtosis coefficients. Potential outliers were identified using box and whisker plots based on standard 1.5 interquartile range and removed before data analysis (only one outlier detected which was a single participant in total leukocyte count). Participant numbers are reported for each variable. Variables with single data points were analyzed using a paired sample t-test or Wilcoxon signed-rank test. Variables with multiple data points were analyzed using a two-way (Trial × Time) repeated-measures ANOVA. Assumptions of homogeneity and sphericity were checked, and adjustments to degrees of freedom were made using the Greenhouse–Geisser correction method when necessary. Significant main effects were further analyzed using post hoc Tukey's HSD test. Ex-GIS data were analyzed using summative accumulation of PRSE vs placebo by the Wilcoxon signed-rank test. Statistical analyzes were performed using SPSS software (version 30.0, IBM SPSS Statistics) with significance set at *p* < 0.05. Visualizing using figures and graphs were performed using GraphPad 10.2.0 (Boston, MA, USA).

## 3. Results

### 3.1. Participant characteristics

**Figure 2.**
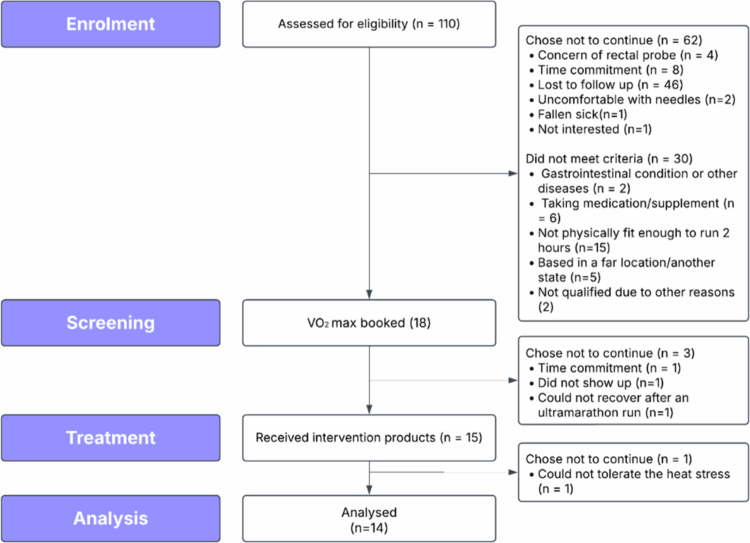
Participant recruitment flow diagram.

Table 1 presents the baseline characteristics of the participants. Fifteen healthy recreational athletes were recruited for the study from May to September 2024. A total of 14 individuals (11 males and 3 females) completed the study after one participant withdrew prematurely due to heat intolerance (Figure 2).

**Table 1.** Participants' baseline characteristics.

Characteristic	Average ± SD
Age (years)	26.3 ± 7.1
Body mass (kg)	72.5 ± 10.1
Height (m)	1.80 ± 0.10
BMI (kg/m^2^)	23.3 ± 2.7
Fat-free mass (kg)	60.6 ± 9.1
Fat mass (kg)	11.8 ± 4.2
Skeletal muscle mass (kg)	29.0 ± 5.2
* $\dot{V}O_{2\max}$ * (mL·kg^−1^·min^−1^)	61.0 ± 7.9
60% $\dot{V}O_{2\max}$	36.6 ± 4.7
Running speed at 60% $\dot{V}O_{2\max}$	9.3 ± 1.2

### 3.2. Thermophysiological strain and hydration status

**Figure 3.**
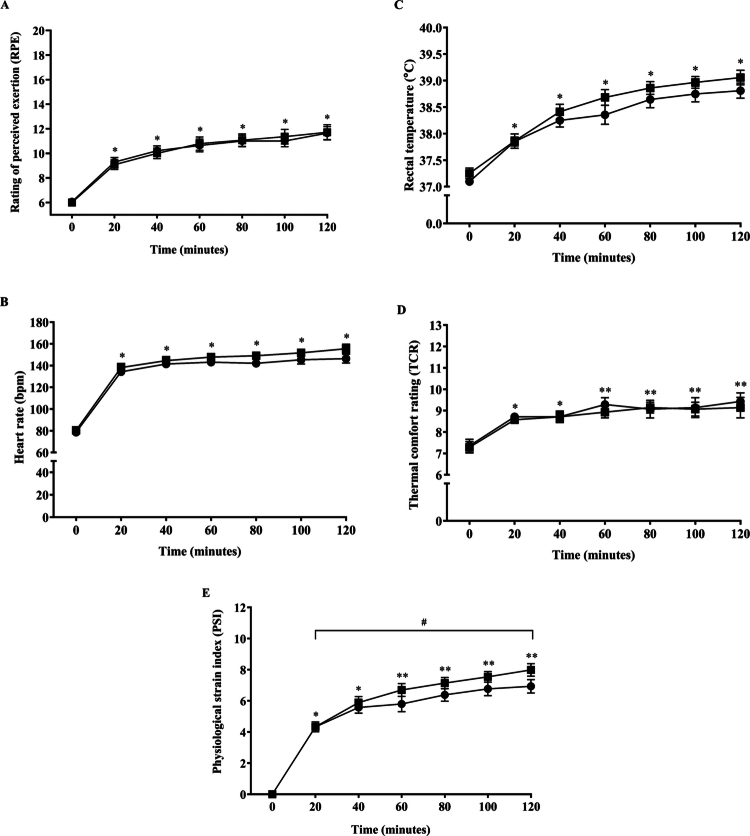
Indicators of thermophysiological strain in response to EHS (2 h running at 60% $\dot{V}O_{2\max}$ in 35 °C ambient temperature and 30% relative humidity) including RPE (A), heart rate (B), rectal temperature (C), thermal comfort rating (TCR) (D) and physiological strain index (PSI) (E) for PRSE (■) and placebo (●). Mean ± SEM (*n* = 14): Main effect of time *p < 0.05 and **p < 0.01 versus pre-EHS. ^#^ *p* < 0.05 between PRSE and placebo trial.

Thermophysiological data are represented in Figure 3. A significant Trial × Time interaction was observed for PSI (*p* = 0.040; Figure 3E) at 60 min (*p* < 0.01) and 120 min (*p* < 0.05) during running, where PRSE exhibited significantly higher values. A significant main effect of time was observed for all thermophysiological measures (*p* < 0.001), with values increasing progressively from pre-EHS to EHS. Between two trials, PRSE exhibited significantly higher T_re_ (*p* = 0.046), and PSI (*p* = 0.010) compared with placebo. No significant Trial × Time interaction was detected for the remaining thermophysiological variables, with no differences between trials (*p* > 0.05). A significant main effect of time was detected (*p* < 0.001) for plasma osmolality (*n* = 11), whereby plasma osmolality significantly decreased post-EHS compared with pre-EHS (*p* < 0.01). No significant Trial × Time interaction was observed and there was no significant difference between PRSE and placebo for plasma osmolality (*p* > 0.05).

### 3.3. Plasma cortisol and blood glucose concentration

**Figure 4.**
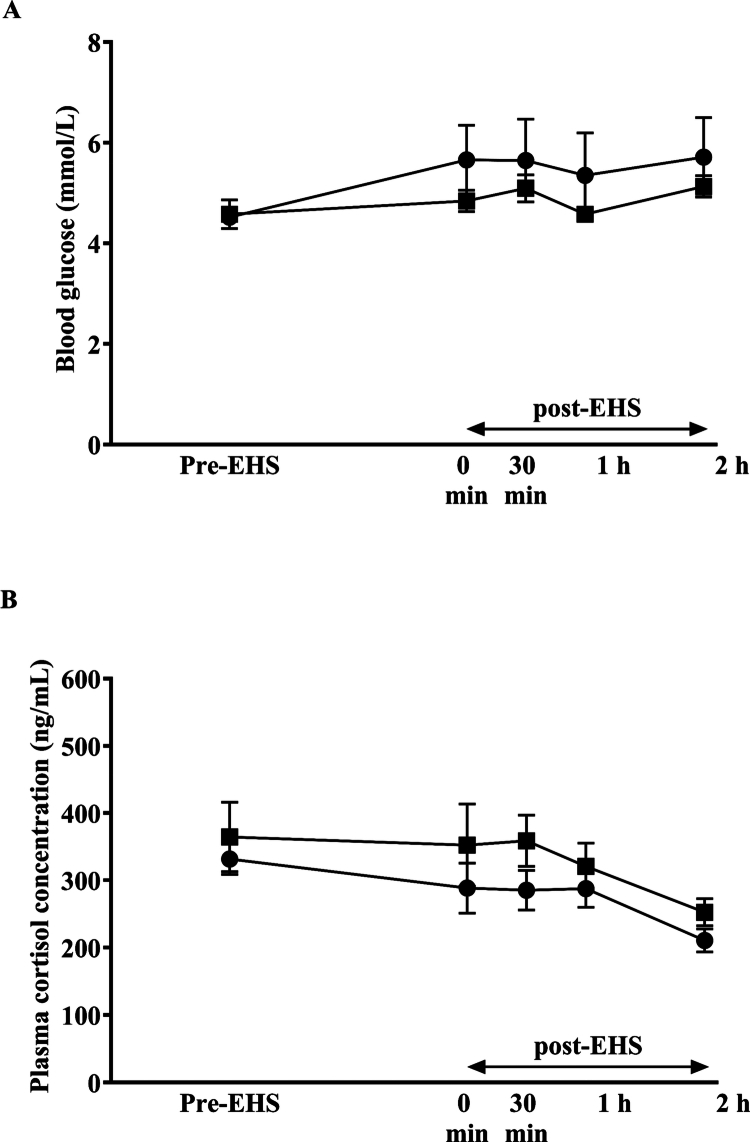
Blood glucose (A) and plasma cortisol (B) concentrations in response to EHS following and PRSE (■) and placebo (●). Mean ± SEM (*n* = 14).

No significant changes were observed (*p* > 0.05) for blood glucose response (Figure 4). A significant main effect of time was detected for cortisol (*p* = 0.039), with concentrations differing over time relative to pre-EHS (Figure 4). However, post-hoc Tukey's test did not identify any time points that differed significantly from pre-EHS. No significant Trial × Time interaction was observed for cortisol, and there was no significant main effect of trial between PRSE and placebo (*p* > 0.05).

### 3.4. Intestinal epithelial integrity

**Figure 5.**
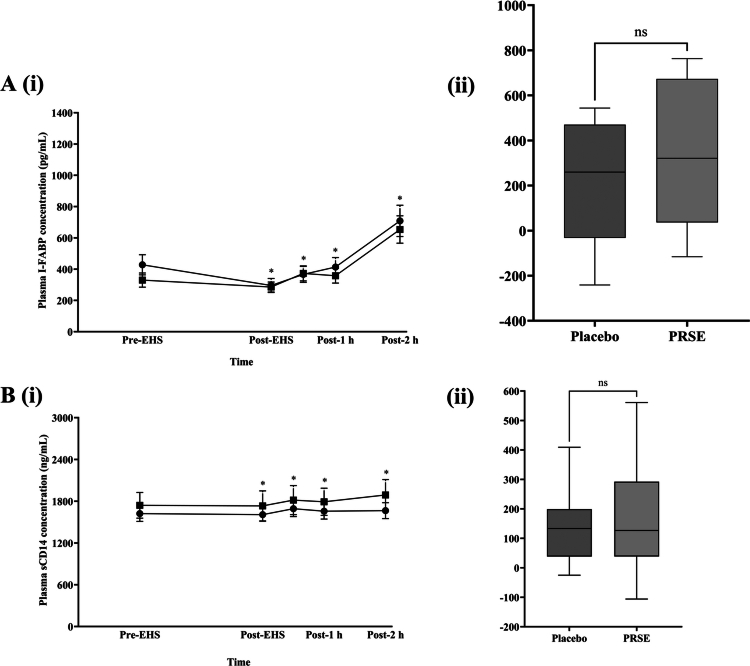
Plasma I-FABP (A) and sCD14 (B) concentrations in response to EHS following PRSE (■) and placebo (●). Mean ± SEM (*n* = 14) response over time (i) and relative Δ box and whisker plot (ii). Main effect of time: * *p* < 0.05 vs pre-EHS detected for both I-FABP and sCD14 at post-2 h. ns = non-significant.

**Figure 6.**
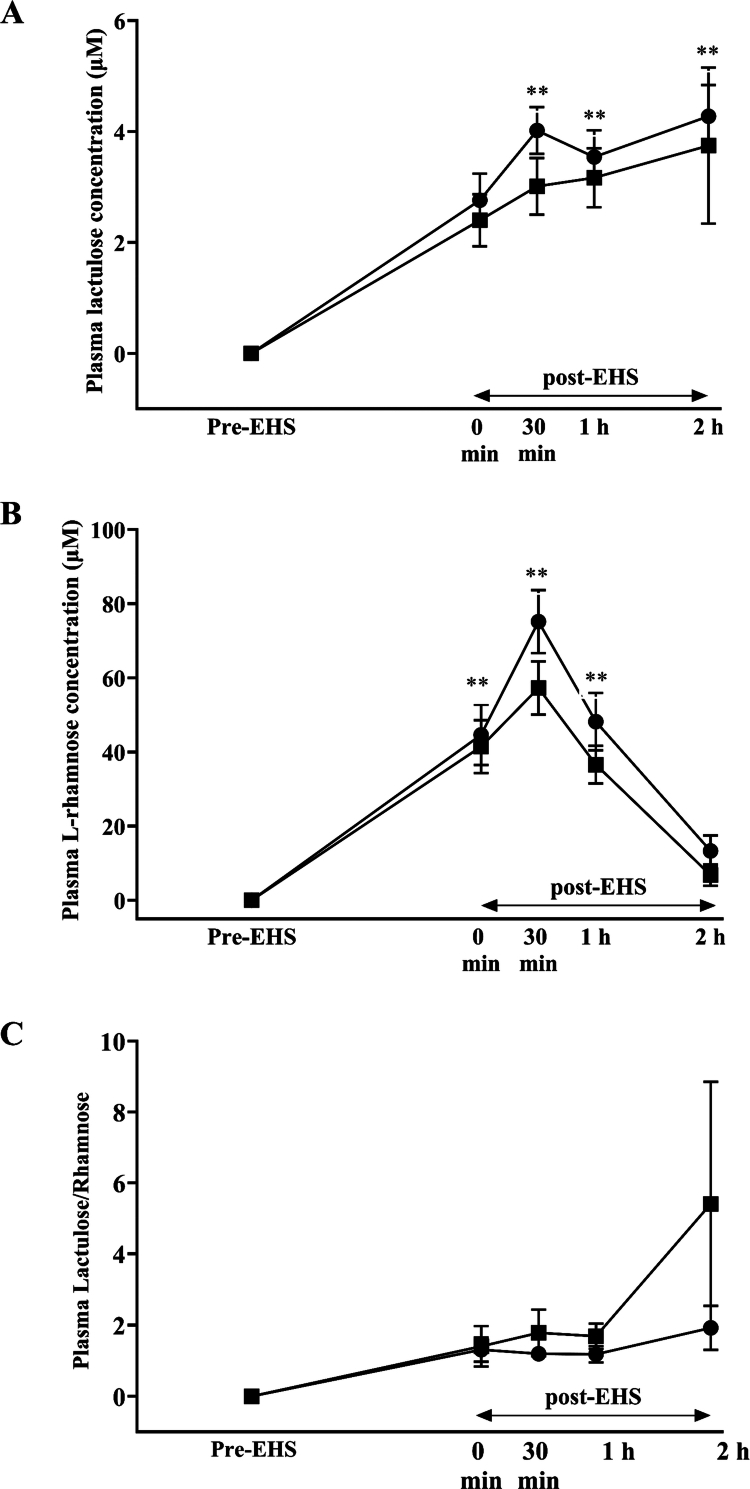
Small intestine permeability measured by the appearance of sugars (lactulose [A], L-rhamnose [B], lactulose/L-rhamnose ratio [C]) in plasma in response to EHS following PRSE intervention (■) and placebo (●). Mean ± SEM (*n* = 14). Main effect of time: *p < 0.001 vs pre-EHS.

No significant Trial × Time interaction was observed for plasma I-FABP, sCD14, or small intestinal permeability assessed using dual sugars (containing lactulose, L-rhamnose, and lactulose/L-rhamnose ratio) (all *p* > 0.05; Figures 5 and 6). A significant main effect of time was observed for plasma I-FABP concentration (*p* = 0.001), plasma sCD14 concentration (*p* = 0.002), and small intestinal permeability assessed by lactulose and L-rhamnose (*p* = 0.001). Plasma I-FABP and sCD14 concentrations increased from pre-EHS to post-2 h exercise (*p* < 0.05), while small intestinal permeability increased from pre-EHS to post-30 min, 1 h, 2 h (*p* < 0.01) time points (Figure 6). Dual sugars were administered during exercise and responses were considered relative to pre-EHS baseline values as done previously [28] (Figure 6). No significant main effect of trial was observed for any of the three biomarkers. At pre-EHS, plasma I-FABP concentrations were significantly lower in PRSE compared with placebo (*p* < 0.05).

### 3.5. Systemic immune responses

No significant Trial × Time interaction was observed for total leukocyte, neutrophils, or lymphocyte counts (*p* > 0.05). Total and differential leukocyte values are reported for *n* = 13 due to one outlier participant. Baseline (pre-EHS) total leukocyte count was 5.4 ± 2.2 × 10⁹·L^−1^ and 4.3 ± 1.3 × 10⁹·L^−1^ for PRSE and placebo, respectively. The relative change from pre-EHS to peak post-EHS was 2.6 ± 1.7 × 10⁹·L^−1^ in PRSE and 3.7 ± 2.3 × 10⁹·L^−1^ in placebo, corresponding to a relative increase of 57% and 94%, respectively. A significant main effect of time was observed for total leukocyte counts (*p* < 0.001); whereby counts increased from pre-EHS to post-1 h (*p* = 0.01) and post-2 h (*p* = 0.05). No significant main effect of trial was observed for total leukocyte counts. Baseline neutrophil counts were 2.8 ± 1.5 × 10⁹·L^−1^ in PRSE and 2.9 ± 3.0 × 10⁹·L^−1^ in placebo, with a relative increase of 2.0 ± 1.3 × 10⁹·L^−1^ (87%) and 2.0 ± 3.6 × 10⁹·L^−1^ (126%), respectively. A significant main effect of time was also detected for neutrophil counts (*p* = 0.001); whereby counts increased from pre-EHS to post-2 h (*p* = 0.01). No significant main effect of trial was observed for neutrophil counts. Baseline lymphocyte counts were 2.2 ± 0.9 × 10⁹·L^−1^ in PRSE and 1.8 ± 0.6 × 10⁹·L^−1^ in placebo, with a relative increasing of 1.0 ± 1.3 × 10⁹·L^−1^ (81%) and 0.8 ± 0.6 × 10⁹·L^−1^ (56%) from pre-EHS to peak post-EHS, respectively. A significant main effect of time was observed for lymphocyte counts (*p* = 0.031); whereby concentrations increased from pre-EHS to post-2 h (*p* = 0.05). No significant main effect of trial was observed for lymphocyte counts.

**Figure 7.**
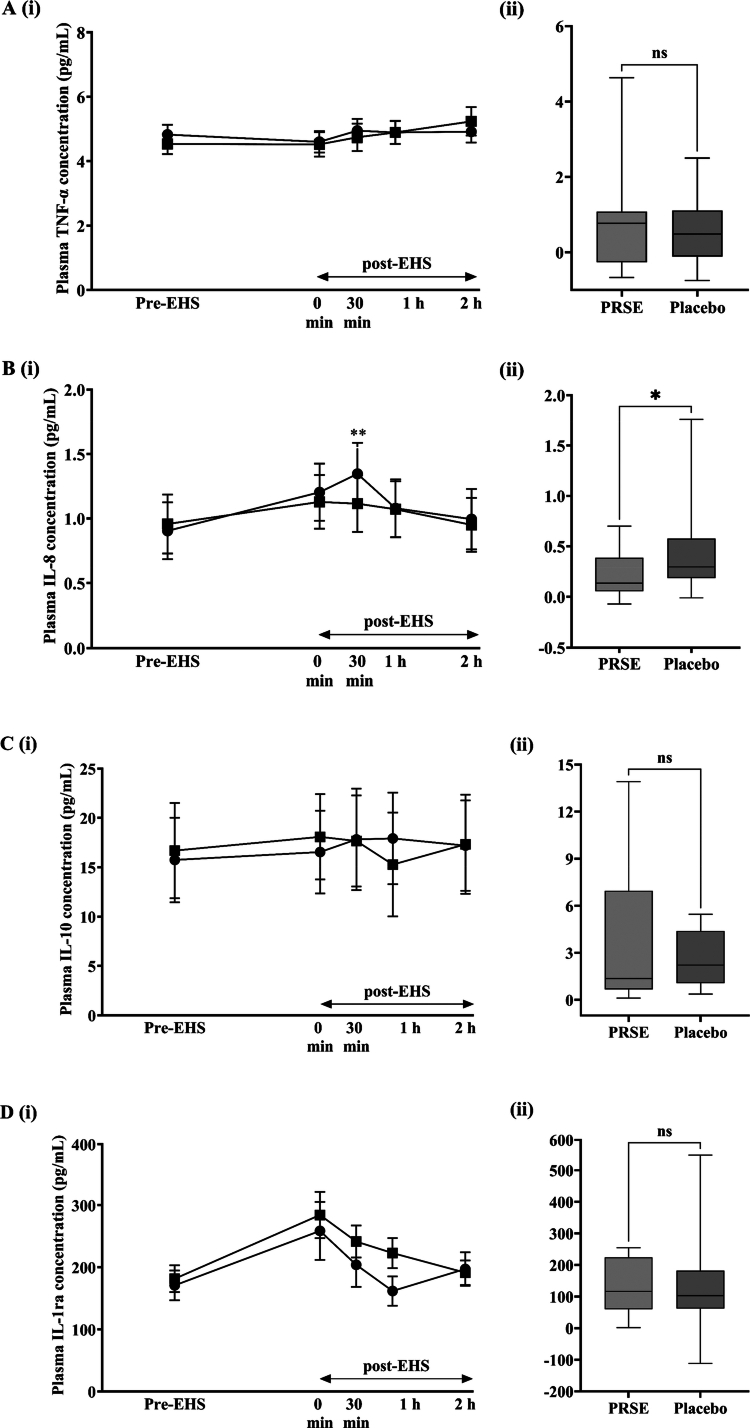
Plasma TNFα (A), IL-18 (B), IL-10 (C), IL-1ra (D) concentrations in response to EHS following PRSE (■) and placebo (●). Mean ± SEM (*n* = 14, except IL-10 *n* = 8) response over time (i) and relative *Δ* box and whisker plot peak (ii). For IL-8 main effect of time: **p < 0.01 vs pre-EHS. Plasma IL-1β and IL-6 concentrations were under minimal detection. ns = not significant.

No significant Trial × Time interaction was observed for plasma cytokine concentrations (all *p* > 0.05), and no significant main effect of trial was detected between PRSE and placebo at any time point (*p* > 0.05; Figure 7). Plasma IL-1β and IL-6 were below the limit of detection in all samples. IL-10 was only detectable in 8 participants (Male: 6, Female 2). A significant main effect of time was observed for plasma IL-8 (*p* < 0.001), whereby concentrations increased from pre-EHS to post-30 min (*p* < 0.05). When the relative change in plasma IL-8 concentration from pre-EHS to peak post-EHS was compared between trials, PRSE demonstrated a significantly smaller increase compared with placebo (*p* = 0.023). No significant difference was observed between trials for SIR-Profile.

### 3.6. Exercise-associated gastrointestinal symptoms (Ex-GIS)

Table 2 represents the incidence and severity of GIS. The prevalence of GIS was 93% in PRSE and 100% in placebo. The incidence and severity of gut discomfort, total-GIS, lower-GIS, upper-GIS, and nausea did not exhibit significant differences across interventions (Table 2). The average severity of Ex-GIS reported was comparatively lower in PRSE in terms of gut discomfort, total-GIS, upper-GIS, and lower-GIS.

**Table 2.** Incidence and severity of gastrointestinal symptoms (GIS) in response to 2 h of exertional-heat stress (EHS) with and without PRSE intervention.

	PRSE		Placebo		*p* value of severity (PRSE vs placebo)
	Incidence (%)	Severity#	Incidence (%)	Severity#	
**Gut discomfort**	NA	9 (2–24)	NA	13 (1–39)	NA
**Total GIS**	93	10 (2–24)	100	15 (1–39)	0.583
**Upper GIS**	79	5 (2–16)	86	7 (1–22)	0.664
Belching	64	3 (2–10)	57	3 (1–13)	0.938
Heartburn	14	0 (2–4)	14	1 (1–14)	>0.999
Upper abdominal bloating	29	1(1–6)	21	1 (2–12)	>0.999
Upper abdominal pain	7	0 (1–1)	7	1 (1–11)	>0.999
Urge to regurgitate	21	1 (1–6)	14	0 (1–1)	>0.999
Regurgitation	0	0 (0)	0	0 (0)	–
Projectile vomiting	0	0 (0)	0	0 (0)	–
**Lower GIS**	50	4 (2–17)	64	6 (2–28)	0.541
Flatulence	29	1 (2–7)	43	2 (2–7)	0.125
Lower abdominal bloating	0	0 (0)	7	0 (5–5)	>0.999
Urge to defecate	29	2 (2–15)	36	3 (1–11)	0.812
Lower abdominal pain	21	1 (2–12)	21	1 (1–12)	>0.999
**Nausea**	14	0 (1–2)	21	1 (1–9)	0.375
Dizziness	14	1 (6–7)	7	0 (5–5)	0.500
Stitch	21	0 (1–2)	21	1 (2–9)	0.312

^#^ Mean of summative accumulation and range of participants reporting incidence (PRSE and placebo, *n* = 14). GIS = gastrointestinal symptoms; NA = not available.

## 4. Discussion

The current study aimed to determine the effects of a PRSE interventions on intestinal epithelial integrity and systemic immune responses to EHS challenge. From our carefully designed protocol, PRSE partially supported our initial hypothesis that it can attenuate intestinal epithelial injury and systemic inflammatory responses to an EHS challenge compared with a carbohydrate-matched placebo, without exacerbating Ex-GIS. PRSE lowered plasma concentrations of pre-EHS I-FABP and post-EHS IL-8 with no effects on other biomarkers. This suggests a modest attenuation of epithelial injury and inflammatory signaling. It is well established that carbohydrate ingestion before and during prolonged exercise attenuates gastrointestinal and inflammatory disturbances to EHS [7,27,28], exerting a dominant protective effect to mitigate EIGS-related biomarkers and systemic inflammation [27,40]. Similar carbohydrate doses in both treatments led to comparable responses observed across the trials under EHS conditions, suggesting a modest additional benefit by PRSE on intestinal epithelial injury (i.e. I-FABP) and systemic inflammatory responses (i.e. IL-8), without exacerbating Ex-GIS, suggesting comparable gastrointestinal tolerance with or without PRSE inclusion.

I-FABP is a sensitive biomarker of enterocyte injury that is rapidly released into circulation from mature small-intestinal enterocytes following epithelial damage [45]. In this study, lower plasma I-FABP concentration was observed at the pre-EHS baseline after two weeks of PRSE supplementation, compared with placebo, suggesting a potential chronic protective effect on epithelial integrity. However, no treatment differences in post-EHS I-FABP responses were observed, likely reflecting the established protective effects of similar carbohydrate ingestion in both treatments during EHS, as reported previously [27]; whereas an additional acute protective effect of PRSE was not observed.

Intestinal epithelial permeability was assessed using the plasma dual sugar absorption test, which provides an index of small intestinal permeability because lactulose and L-rhamnose are nonmetabolized [46]. Differences between plasma and urine-based dual sugar methodologies should be considered when interpreting gastrointestinal permeability findings across studies. Urinary dual sugar assessment, as used in previous work by Snipe et al. [27] provides a cumulative index of sugar recovery over an extended collection period and has demonstrated sensitivity to acute exercise-induced gastrointestinal perturbations. In contrast, Houghton et al. [28] reported that plasma dual sugar measurements obtained at different postexercise time points detected transient increases in permeability following EHS that were not observed using 5-h urinary collection from the same exercise trials. Houghton et al. [28] suggested that differing appearance kinetics between lactulose and rhamnose may allow short-lived permeability changes to be more readily detected in plasma at specific time points; whereas, cumulative urinary recovery may reflect a broader integrated response across the collection period. However, plasma concentrations also reflect systemic clearance kinetics (i.e. affected by individual metabolism, renal function, and excretion rate) in addition to gastrointestinal passage [28]. Therefore, plasma and urine-based methodologies may provide different temporal perspectives of exercise-induced gastrointestinal permeability responses.

Increased intestinal epithelial permeability facilitates the translocation of pathogenic luminal content, including bacterial endotoxins, which can be indirectly assessed via plasma soluble CD14 (sCD14), a co-receptor for lipopolysaccharides [47]. In this case, EHS increased intestinal permeability and plasma sCD14 concentration post-EHS, yet no differences were observed between PRSE and placebo. Similar carbohydrate ingestion across both treatments may have blunted the acute changes in permeability and endotoxin translocation during EHS, with no additional effect from PRSE [27,28].

Increased intestinal permeability and translocation of pathogenic luminal content into circulation during EHS promotes systemic immune activation, reflected by elevations in circulating leukocytes and inflammatory mediators [48]. In the current study, total and differential leukocyte counts increased in response to EHS, consistent with previous reports of exercise- and heat-induced leukocytosis [21,49,50]. No differences were observed between PRSE and placebo, indicating that PRSE did not alter the magnitude of systemic cellular immune response. Baseline leukocyte values and the magnitude of exercise-induced increases were comparable to those reported in previous EIGS studies using similar experimental models [38].

Systemic cytokine responses were generally modest, with no between-trial differences observed for most cytokines assessed, aligning with previous laboratory-based EHS studies reporting mild cytokinemia despite substantial physiological strain [27,39,43,51]. This outcome further emphasized the impact of real-life EHS (e.g. multi-stage ultramarathon competition) on substantially exacerbating systemic inflammatory responses and warranting caution when interpreting or translating laboratory-based immune response findings into real-world practice [52]. In addition, carbohydrate-rich beverages (both placebo and PRSE) seem to have abolished IL-1β and IL-6 response, similar to prior studies [27]. Notably, PRSE attenuated the EHS-induced increase in plasma IL-8 response, suggesting its influence on neutrophil recruitment (i.e. functional aspects) and the amplification of inflammatory signaling regulated by multiple transcription factors, including NF-κB and activator protein-1 (AP-1) [29]. The attenuated plasma IL-8 response may be explained by (poly)phenol-mediated modulation of transcriptional pathways regulating IL-8 expression. IL-8 is regulated by multiple transcription factors, including NF-κB and activator protein-1 (AP-1). Several hours of treatment with freeze-dried sugarcane stem extracts (extracted with 20% v/v ethanol) have been reported to reduce IL-8 secretion by inhibiting NF-κB activity in human intestinal Caco-2 cells [53]. Another *in vitro* study using a human colon carcinoma cell model demonstrated that whole dried sugarcane extract (extracted by 52% ethanol v/v in an ultrasonic water bath at 60 °C for 30 min) suppressed AP-1 activity, via reduced c-Jun Ser63 phosphorylation, which may be the basis for IL-8 attenuation [29]. Similarly, PRSE selectively influenced IL-8 responses in this study despite the absence of broader cytokine changes. It may be worth exploring the effects of PRSE in the downstream of IL-8-mediated inflammatory signaling pathways in the future.

Ex-GIS are a common consequence of EHS, reflecting disturbances to gastrointestinal integrity and function (i.e. impact on feeding tolerance) [7]. The use of a validated and reliability-checked exercise-specific gastrointestinal symptoms assessment tool in this study enabled robust characterization of symptom type, incidence, and severity [38,54]. Ex-GIS incidence in response to EHS was high in both treatments (PRSE and placebo), with no differences between PRSE and placebo. However, Ex-GIS severity average reported was lower in the PRSE trial across multiple domains, including gut discomfort and total-GIS. These findings align with previous research showing that nutritional interventions more commonly influence GIS severity rather than incidence during exercise in the heat [25,26]. Similarly, reviews of (poly)phenol intake in gastrointestinal disorders reported reductions in symptom severity despite limited evidence for clinical value [55].

A key strength of this study is the application of a physiologically valid EHS model that elicited substantial physiological, thermoregulatory, gastrointestinal, endocrine, and immunological strain. The comprehensive assessment of intestinal injury, permeability, bacterial endotoxin translocation, leukocyte counts, inflammatory responses, and Ex-GIS provides an integrated evaluation of the EHS on pathophysiology of EIGS. The crossover design further strengthened internal validity by minimizing interindividual variability, which is particularly important for biomarkers with high biological variability [38,56].

Several limitations should be acknowledged. Although most thermophysiological parameters were comparable between trials, rectal temperature and physiological strain index were modestly elevated during PRSE treatment at specific time points. This introduces interpretative complexity, as increased thermal strain may independently exacerbate perturbations of the gastrointestinal epithelium. However, the magnitude of these differences was modest and remained within ranges typically reported in controlled EHS protocols, suggesting no immediate safety concern under the present experimental conditions.

While the underlying mechanism cannot be definitively established, this observation may reflect subtle modulation of metabolic or autonomic responses during exertional heat stress. (Poly)phenols have been reported to influence cellular energy metabolism and thermogenic pathways [57] and alterations in substrate utilization and sympathetic nervous system activity [58,59] both of which may influence heat production during exercise. However, these mechanisms were not directly assessed in the present study and therefore remain speculative. Importantly, despite the modest elevation in rectal temperature, no exacerbation of intestinal epithelial injury, permeability, or systemic inflammatory responses was observed in the PRSE condition relative to placebo. In contrast, plasma I-FABP concentrations were lower at pre-EHS and IL-8 responses were attenuated postexercise, potentially indicating preserved epithelial integrity under conditions of comparable or marginally increased physiological strain. Nevertheless, this observation warrants cautious interpretation and further investigation to determine its reproducibility and underlying mechanisms.

The sample size for this study was determined a priori based on the primary outcome, for which adequate statistical power was achieved. However, it is acknowledged that this sample size may have been insufficient to detect smaller, yet potentially meaningful, effects in secondary outcomes. The use of nonacclimatized participants was intended to enable controlled evaluation of physiological perturbations under standardized conditions, without the variability introduced by differing levels of prior heat adaptation. Nevertheless, caution is warranted when extrapolating these findings to real-world settings involving heat-acclimatized populations, and future studies should investigate whether similar responses are observed following acclimatization. The absence of a water-only placebo limits insight into the independent effects of carbohydrate and PRSE; however, inclusion of an additional experimental arm would have increased participant burden within an already demanding protocol. Importantly, water-only responses during comparable EHS protocols have been well characterized in previous work [27]. Baseline variability in plasma I-FABP concentration between trials also complicates the interpretation of post-EHS responses, although it may indicate a chronic effect of PRSE on epithelial integrity. Finally, the generalizability of the observed cytokine responses is limited by the low detectability of some cytokines in this healthy endurance-trained cohort.

In conclusion, two weeks of PRSE supplementation may offer modest and targeted benefits to intestinal epithelial integrity and systemic inflammatory regulation in response to EHS, without further exacerbating gastrointestinal symptoms. Further research is required to determine whether these effects are applicable in wider populations, especially within occupational health (e.g. military, mining, agriculture, construction, and fieldwork), and those exposed to hot ambient conditions while undertaking physically active occupations.
